# Author Correction: Single-nucleus multi-omics of human stem cell-derived islets identifies deficiencies in lineage specification

**DOI:** 10.1038/s41556-023-01219-4

**Published:** 2023-08-09

**Authors:** Punn Augsornworawat, Nathaniel J. Hogrebe, Matthew Ishahak, Mason D. Schmidt, Erica Marquez, Marlie M. Maestas, Daniel A. Veronese-Paniagua, Sarah E. Gale, Julia R. Miller, Leonardo Velazco-Cruz, Jeffrey R. Millman

**Affiliations:** 1 Division of Endocrinology, Metabolism and Lipid Research, Washington University School of Medicine, MSC 8127-057-08, St. Louis, MO USA; 2 Department of Biomedical Engineering, Washington University in St. Louis, St. Louis, MO USA

**Keywords:** Embryonic germ cells, Type 1 diabetes, Stem-cell biotechnology

Correction to: *Nature Cell Biology* 10.1038/s41556-023-01150-8. Published online 15 May 2023.

In the version of this article initially published, the Fig. 1c heatmap for verifying the identified cell types with key gene markers presented the *x*-axis *DLK1* and *GCG* gene labels, respectively, in the wrong order. The error does not affect the results or conclusions in the study. The figure is now updated in the HTML and PDF versions of the article.

