# Supplementary material for: Single-nucleus multi-omics of human stem cell-derived islets identifies deficiencies in lineage specification
Source: Nat Cell Biol. 2023 May 15;25(6):904–16. doi: 10.1038/s41556-023-01150-8 (PMC10264244; doi:10.1038/s41556-023-01150-8)
Supplement: Supplementary file 1 — Supplementary Figs. 1–5. [file 41556_2023_1150_MOESM1_ESM.pdf]

nature cell biology

Resource

https://doi.org/10.1038/s41556-023-01150-8

Single-nucleus multi-omics of human stem cell-derived islets identifies deficiencies in lineage specification

In the format provided by the authors and unedited

Supplementary Figure 1

Tape station figures of ATAC libraries

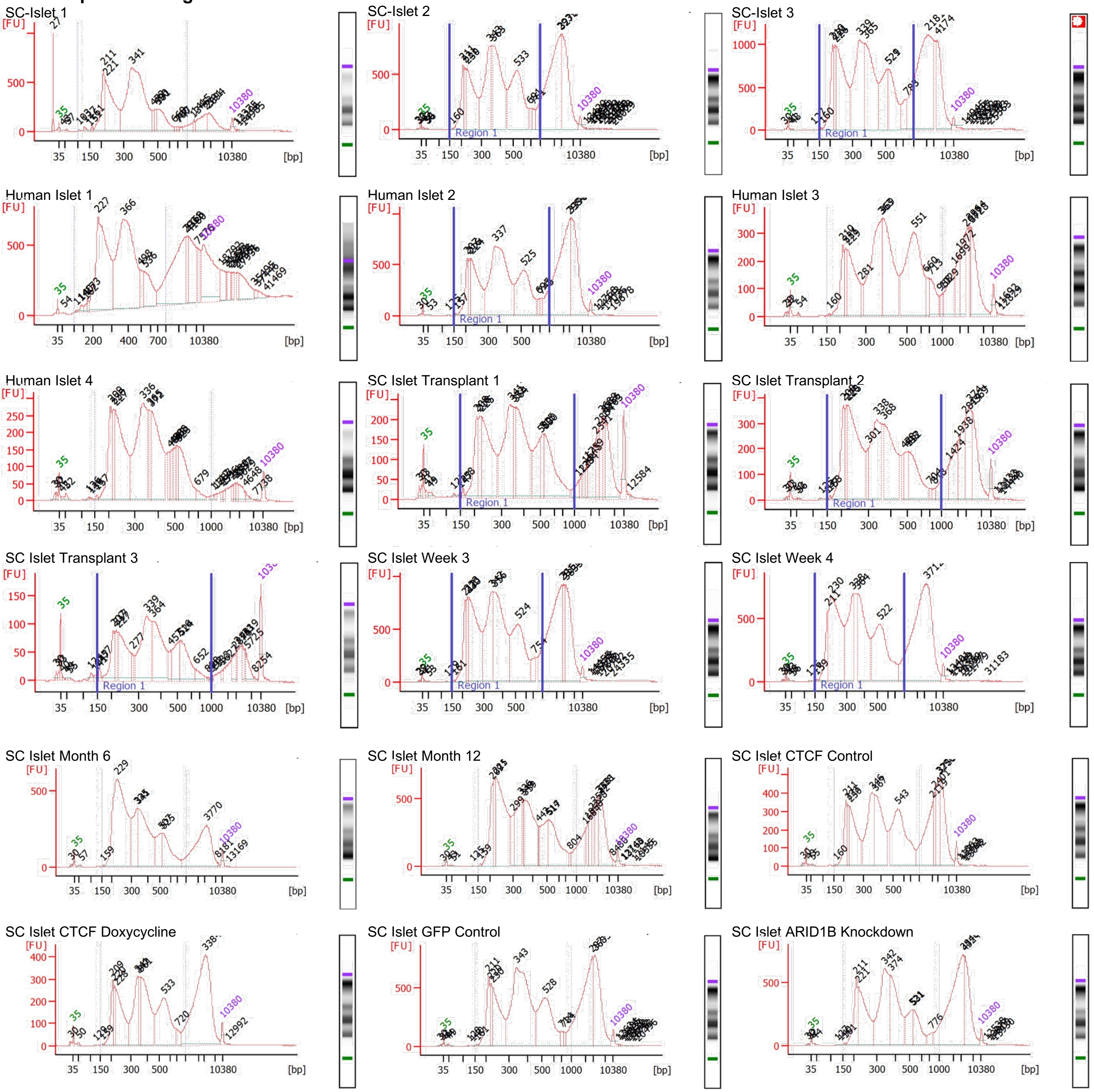

Supplementary Figure 1. Tape station figures of single nuclei ATAC libraries for all multiome samples.

Supplementary Figure 2

Cell exclusion strategy for single-cell multiome analysis

SC-Islet and human islet

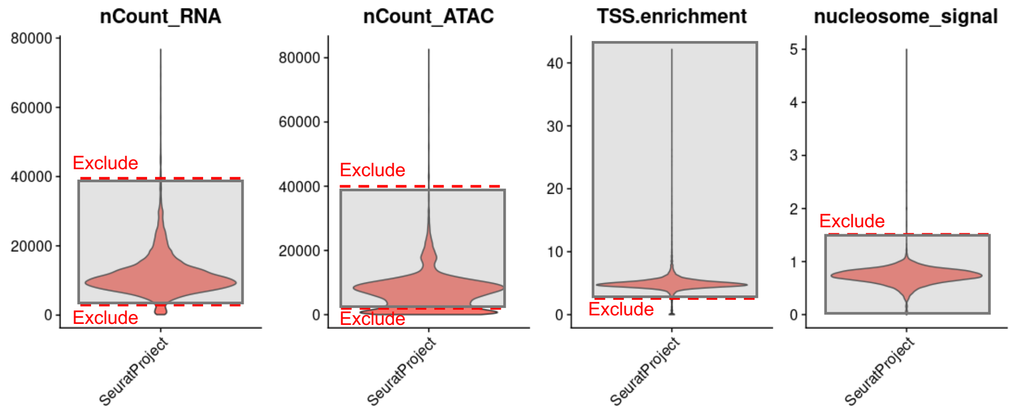

Transplanted SC-islet

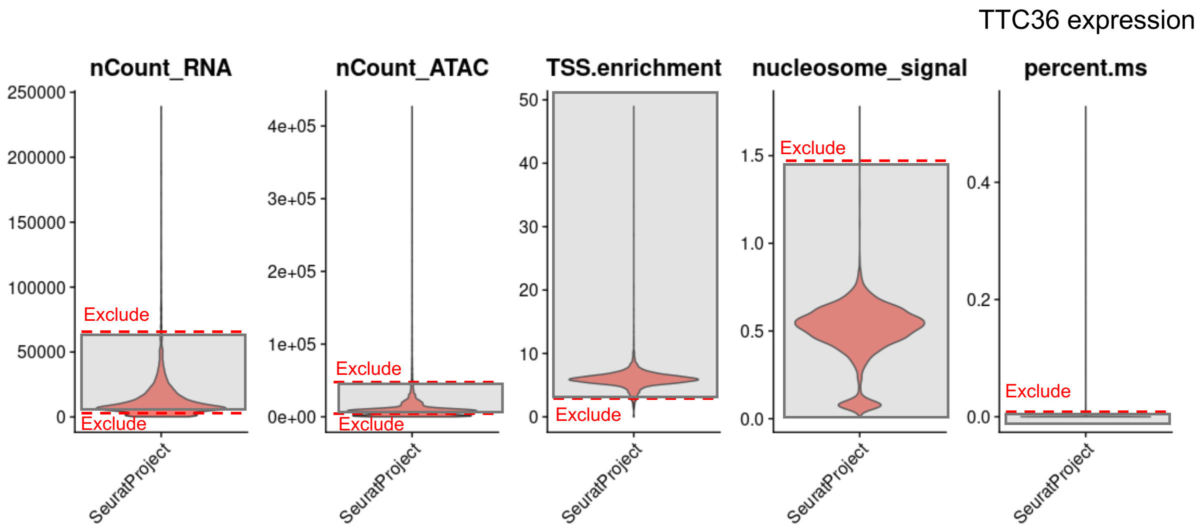

Supplementary Figure 2. Cell exclusion strategy to remove low quality cells for the single-nuclei multiome analysis. Ranges outside the shaded regions were excluded.

Supplementary Figure 3

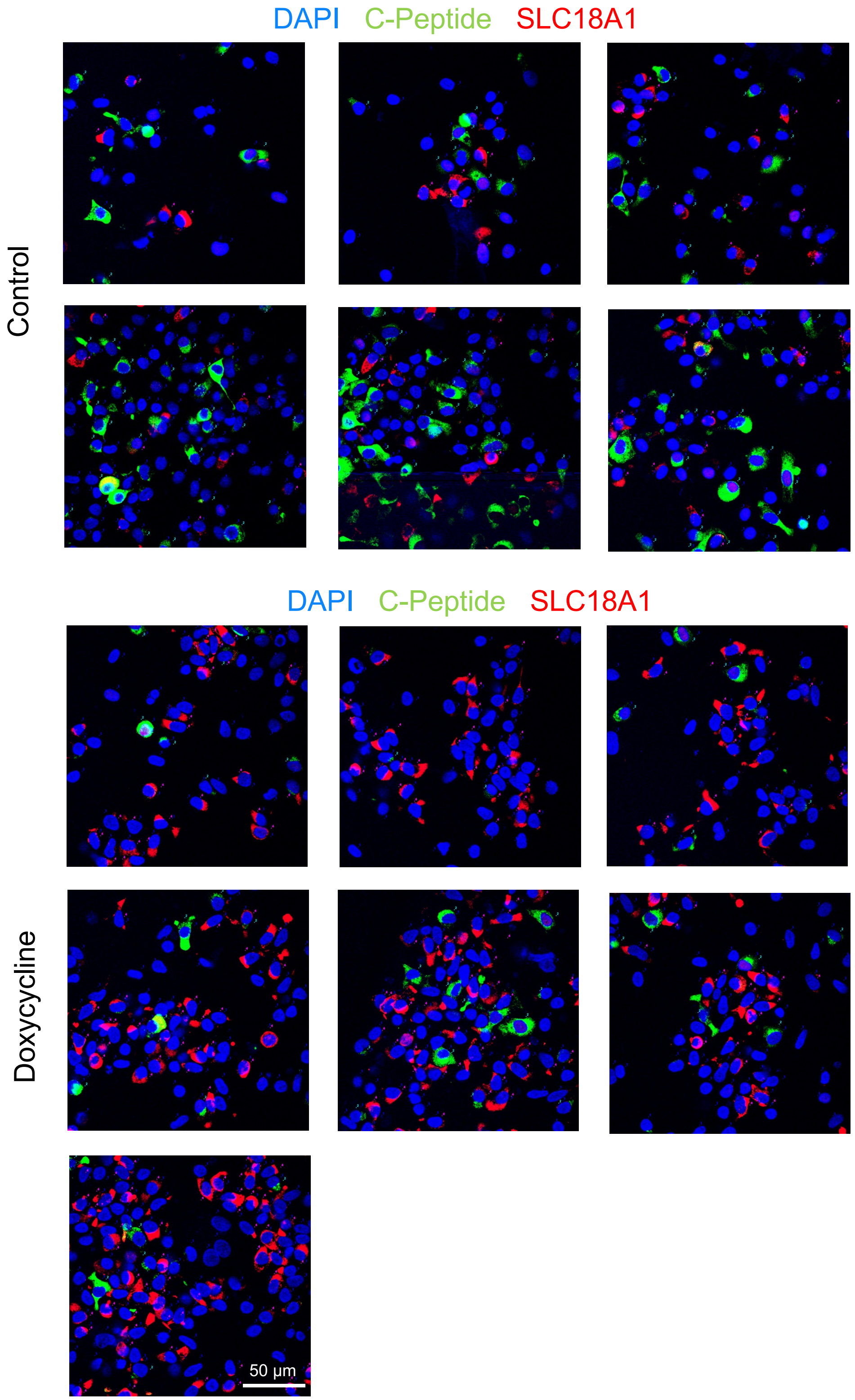

Supplementary Figure 3. Immunofluorescent images showing β-cell marker C-peptide and EC-cell marker SLC18A1 stains from individual samples used to quantify β-cell and EC-cell composition.

Supplementary Figure 4

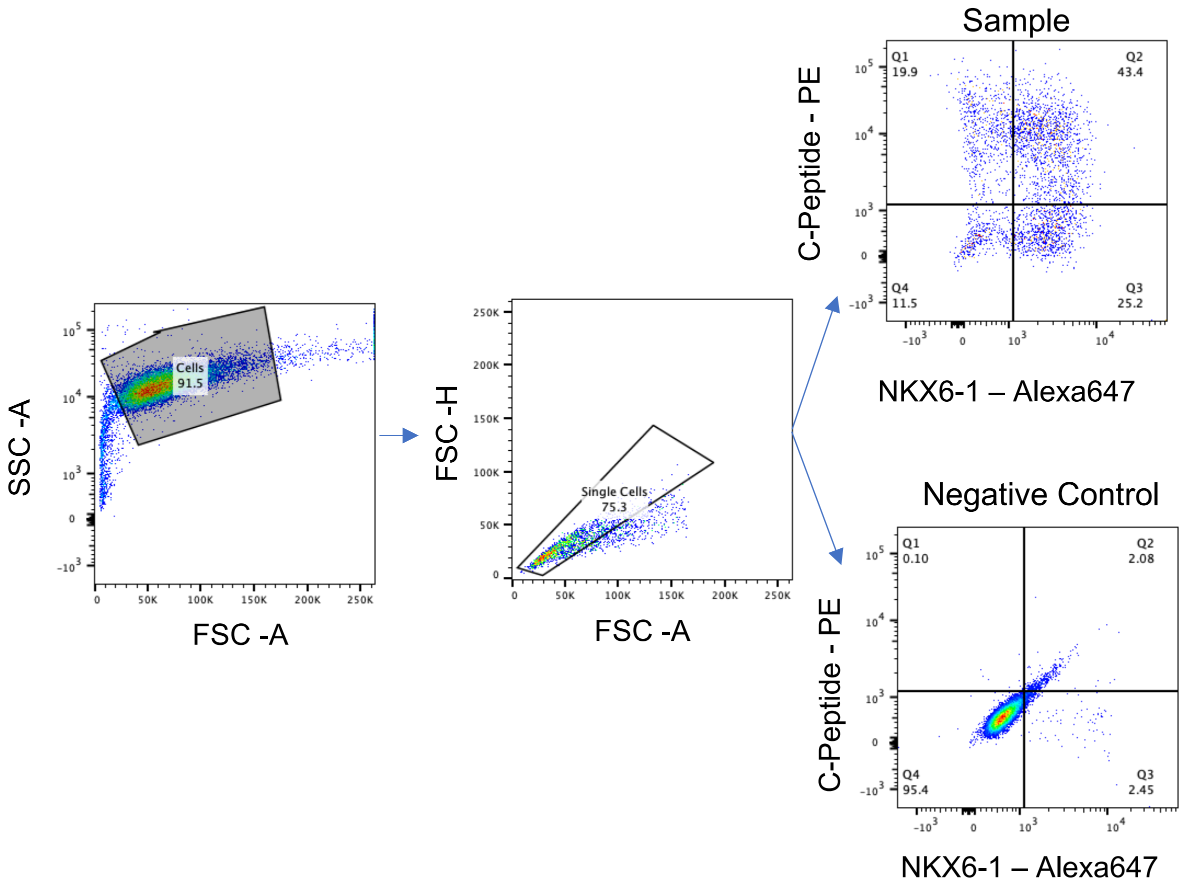

Supplementary Figure 4. Gating strategy used for flow cytometry analysis.

Supplementary Figure 5

ARID1B shRNA

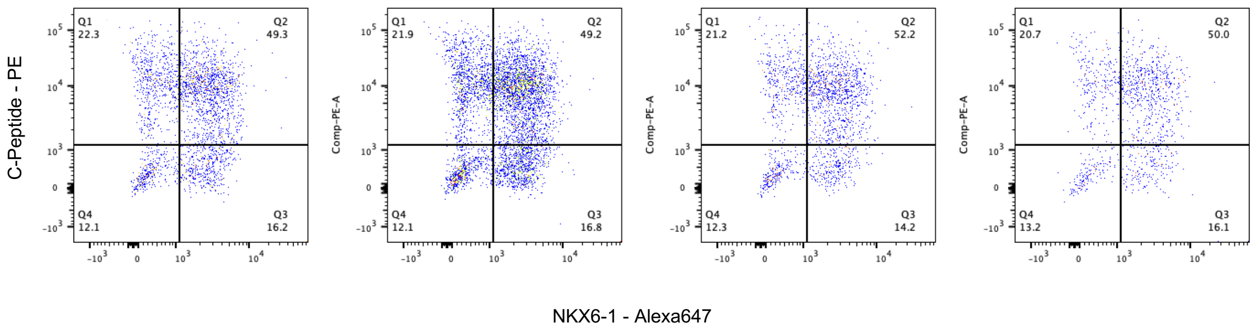

Control

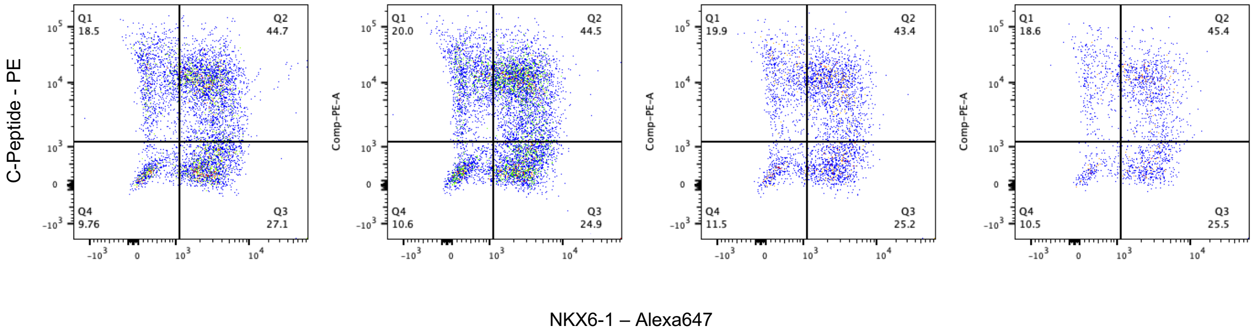

Negative Control Sample

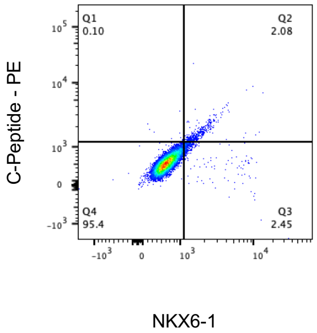

Supplementary Figure 5. Individual flow cytometry graphs showing stem-cell derived β-cell makers NKX6-1 and C-Peptide from differentiated SC-islets with and without ARID1B knockdown.
