# Supplementary material for: Single-nucleus multi-omics of human stem cell-derived islets identifies deficiencies in lineage specification
Source: Nat Cell Biol. 2023 May 15;25(6):904–16. doi: 10.1038/s41556-023-01150-8 (PMC10264244; doi:10.1038/s41556-023-01150-8)
Supplement: Supplementary file 2 — Reporting Summary [file 41556_2023_1150_MOESM2_ESM.pdf]

nature portfolio

Corresponding author(s): Jeffrey R. Millman

Last updated by author(s): Apr 13, 2023

Reporting Summary

Nature Portfolio wishes to improve the reproducibility of the work that we publish. This form provides structure for consistency and transparency in reporting. For further information on Nature Portfolio policies, see our Editorial Policies and the Editorial Policy Checklist.

Statistics

For all statistical analyses, confirm that the following items are present in the figure legend, table legend, main text, or Methods section.

n/a	Confirmed	
☐	☒	The exact sample size (*n*) for each experimental group/condition, given as a discrete number and unit of measurement
☐	☒	A statement on whether measurements were taken from distinct samples or whether the same sample was measured repeatedly
☐	☒	The statistical test(s) used AND whether they are one- or two-sided<br>*Only common tests should be described solely by name; describe more complex techniques in the Methods section.*
☒	☐	A description of all covariates tested
☐	☒	A description of any assumptions or corrections, such as tests of normality and adjustment for multiple comparisons
☐	☒	A full description of the statistical parameters including central tendency (e.g. means) or other basic estimates (e.g. regression coefficient) AND variation (e.g. standard deviation) or associated estimates of uncertainty (e.g. confidence intervals)
☐	☒	For null hypothesis testing, the test statistic (e.g. *F*, *t*, *r*) with confidence intervals, effect sizes, degrees of freedom and *P* value noted<br>*Give P values as exact values whenever suitable.*
☒	☐	For Bayesian analysis, information on the choice of priors and Markov chain Monte Carlo settings
☒	☐	For hierarchical and complex designs, identification of the appropriate level for tests and full reporting of outcomes
☐	☒	Estimates of effect sizes (e.g. Cohen's *d*, Pearson's *r*), indicating how they were calculated

Our web collection on statistics for biologists contains articles on many of the points above.

Software and code

Policy information about availability of computer code

Data collection	Single-cell sequencing data was collected using the 10X Chromium and Illumina NovaSeq 6000. Flow cytometry data was collected using the LSR Fortessa and BD FACSDiva software. qPCR data was collected from the QuantStudio6 Pro using Design & Analysis 2.6.0. Fluorescent images were collected from the Zeiss Cell Discoverer confocal 7.
Data analysis	Single-cell multiomics sequencing data was analyzed using Cell Ranger ARC v2.0, and R studio 1.3.1093 (R v 4.03). We used packages Seurat 4.01, Signac 1.3.0, Monocle3 1.0, and chromVAR 1.12.0 . Flow cytometry was analyzed using FlowJo v10.8.1.

For manuscripts utilizing custom algorithms or software that are central to the research but not yet described in published literature, software must be made available to editors and reviewers. We strongly encourage code deposition in a community repository (e.g. GitHub). See the Nature Portfolio guidelines for submitting code & software for further information.

Data

Policy information about availability of data

All manuscripts must include a data availability statement. This statement should provide the following information, where applicable:

- Accession codes, unique identifiers, or web links for publicly available datasets
- A description of any restrictions on data availability
- For clinical datasets or third party data, please ensure that the statement adheres to our policy

Sequencing data that support the findings of this study have been deposited in the Gene Expression Omnibus (GEO) under accession code GSE199636. Source data

are provided with this study. GRCh38 human genome (Schneider et al.), MSigDB (Subramanian et al.) and JASPAR2020 (Fornes et al.) databases were used. All other data supporting the findings of this study are available from the corresponding author on reasonable request.

Human research participants

Policy information about studies involving human research participants and Sex and Gender in Research.

Reporting on sex and gender	Three male and one female donor for human islets were used.
Population characteristics	Donors for human islets ranged from 19-31.6 BMI and 18-68 years of age.
Recruitment	Prodo laboratories were involved in the recruitment of donors. We were not part of this process.
Ethics oversight	Prodo laboratories were involved in the recruitment of donors. We were not part of this process.

Note that full information on the approval of the study protocol must also be provided in the manuscript.

Field-specific reporting

Please select the one below that is the best fit for your research. If you are not sure, read the appropriate sections before making your selection.

☒ Life sciences ☐ Behavioural & social sciences ☐ Ecological, evolutionary & environmental sciences

For a reference copy of the document with all sections, see nature.com/documents/nr-reporting-summary-flat.pdf

Life sciences study design

All studies must disclose on these points even when the disclosure is negative.

Sample size	Sample sizes for single-nuclei multiome were determined based on 10x genomics recommended instructions. Target sequencing cell number was 10000, unless samples have lower cell count. Sample sizes in other experiments were based on previous experiences and studies with citations included in this study. No statistical methods were used to pre-determine sample sizes but our sample sizes are similar to those reported in previous publications (Pagliuca et al., Veres et al., Augsornworawat et al., Chiou et al., Hogrebe et al.).
Data exclusions	In single-nuclei mutiomics sequencings, low quality sequenced cells were removed with low RNA counts (nCount_RNA < 1000) and low ATAC counts (nCount_ATAC < 1000); excessively high RNA counts (ranging > 40000 – 50000) and excessively high ATAC counts (ranging > 40000 – 50000); nucleosome signal > 1.25, and TSS enrichment < 2. Mouse cells were excluded by cells expressing TTC36.
Replication	All results presented were replicated across multiple independent differentiations at least 3 times. Human islet datasets were obtained from different donors. In vitro experiments have been validated and performed by multiple personnels. Transplant datasets came from multiple mice.
Randomization	Randomization is not applicable in this study because there are no human participants and no clinical trials.
Blinding	Data collection and analysis were not performed blind to the conditions of the experiments. Blinding was not used in this study because there are no human participants that is subject to bias.

Reporting for specific materials, systems and methods

We require information from authors about some types of materials, experimental systems and methods used in many studies. Here, indicate whether each material, system or method listed is relevant to your study. If you are not sure if a list item applies to your research, read the appropriate section before selecting a response.

Materials & experimental systems

n/a	Involved in the study	
☐	☒	Antibodies
☐	☒	Eukaryotic cell lines
☒	☐	Palaeontology and archaeology
☐	☒	Animals and other organisms
☒	☐	Clinical data
☒	☐	Dual use research of concern

Methods

n/a	Involved in the study	
☒	☐	ChIP-seq
☐	☒	Flow cytometry
☒	☐	MRI-based neuroimaging

Antibodies

Antibodies used	C-peptide (DSHB GN-ID4-S; 1:300)<br>RRID: AB_2255626<br>GN-ID4 was deposited to the DSHB by Madsen, O.D. (DSHB Hybridoma Product GN-ID4)<br><br>IAPP (Sigma Aldrich; PA5-84142; 1:300)<br>RRID: AB_2806862<br><br>NKX6-1 (DSHB; F55A12-S; 1:100)<br>RRID: AB_532379<br>F55A12 was deposited to the DSHB by Madsen, O.D. (DSHB Hybridoma Product F55A12)<br><br>SLC18A1 (Sigma Aldrich; HPA063797; 1:300)<br>Manufacturer validation: immunohistochemistry. Additional validation by the Human Protein Atlas (HPA) project.<br><br>Secondary Antibodies Company Part #<br>Anti-rat alexa fluor 488 (Invitrogen, cat. no. A21208; RRID: AB_141709)<br>Anti-rabbit alexa fluor 488 (Invitrogen, cat. no. A21206; RRID: AB_2535792)<br>Anti-mouse alexa fluor 594 (Invitrogen, cat. no. A21203; RRID: AB_141633)<br>Anti-rabbit alexa fluor 647 (Invitrogen, cat. no. A31573; RRID: AB_2536183)<br>Anti-rat PE (Jackson ImmunoResearch, cat. no. 712-116-153; RRID: AB_2340657)
Validation	All antibodies used have been validated on the supplier web page and used previously in other literatures. (Hogrebe et al., Nature 16 9 2021; Augsornworawat et al., Cell reports 32 8 2020; Veres et al., Nature 569 7756 2019; Pagliuca et al., Cell 159 2 2014)

Eukaryotic cell lines

Policy information about cell lines and Sex and Gender in Research

Cell line source(s)	HUES8 hESC line was provided by Douglas Melton (Harvard University)<br>H1 hESC line was provided by Lindy Barrett (Broad Institute) with permission from WiCell
Authentication	All lines have been authenticated with DNA fingerprinting.
Mycoplasma contamination	Hues 8 and H1 hESC tested negative for mycoplasma as tested by Washington Universicty GEiC.
Commonly misidentified lines<br>(See ICLAC register)	No cell lines listed used.

Animals and other research organisms

Policy information about studies involving animals; ARRIVE guidelines recommended for reporting animal research, and Sex and Gender in Research

Laboratory animals	7 week old, male, NOD.Cg-Prkdcscid Il2rgtm1wjl/SzJ (NSG) mice (Jackson Laboratories; 005557). Mice were housed in an ambient facility with 30-70% humidity and a 12-hr light/dark cycle and were fed a chow diet.
Wild animals	No wild animals used in this study.
Reporting on sex	In vivo experiments involved male mice only.
Field-collected samples	No field-collected samples in this study.
Ethics oversight	Animal studies were performed by unblinded individuals in accordance with the Washington University International Care and Use Committee (IACUC) guidelines (Approval 21-0240).

Note that full information on the approval of the study protocol must also be provided in the manuscript.

Flow Cytometry

Plots

Confirm that:

☒ The axis labels state the marker and fluorochrome used (e.g. CD4-FITC).

☒ The axis scales are clearly visible. Include numbers along axes only for bottom left plot of group (a 'group' is an analysis of identical markers).

☒ All plots are contour plots with outliers or pseudocolor plots.

☒ A numerical value for number of cells or percentage (with statistics) is provided.

Methodology

Sample preparation	Cells were single-cell dispersed by washing with PBS and adding 0.2 mL TrypLE/cm2 for 10minutes at 37C. Cells were fixed for 30 minutes in 4% PFA at 4C followed by incubation in ICC solution for 45 minutes in 4C. Primary antibodies were prepared in ICC solution and incubated on cells overnight at 4C. The following day, cells were washed with ICC solution and incubated for 2hr in secondary antibody at 4C. Cells were washed twice with PBS and filtered before running on an LSR Fortessa flow cytometer (BD Bioscience). FlowJo was used for analysis.
Instrument	LSR Fortessa flow cytometer (BD Bioscience)
Software	FlowJo v10.8.1 was used for analysis
Cell population abundance	Flow cytometry populations were greater than 30%.
Gating strategy	Gating strategy example is shown in Supplementary Information.

☒ Tick this box to confirm that a figure exemplifying the gating strategy is provided in the Supplementary Information.
